# What factors best explain attitudes to snow leopards in the Nepal Himalayas?

**DOI:** 10.1371/journal.pone.0223565

**Published:** 2019-10-23

**Authors:** Jonathan H. Hanson, Maurice Schutgens, Nigel Leader-Williams

**Affiliations:** 1 Department of Geography, University of Cambridge, Cambridge, United Kingdom; 2 Independent Researcher, Port Shepstone, South Africa; University of Bucharest, ROMANIA

## Abstract

The snow leopard *Panthera uncia* is a vulnerable wild felid native to mountainous regions of 12 Asian countries. It faces numerous overlapping threats, including killings by herders retaliating against livestock losses, the illegal wildlife trade, loss of prey and habitat, infrastructure, energy and mining developments, and climate change. The species ranges over large territories that often lie outside of protected areas (PA), so coexistence with human populations across its range is key to its persistence. Human attitudes to snow leopards may be an important factor to consider in reducing overlapping threats to this species. However, this nexus has not been widely studied to date. Attitudes to snow leopard conservation, including actors and interventions, may also be a significant aspect of coexistence. These have also received limited empirical attention. This study therefore explored human attitudes to snow leopards and to snow leopard conservation, the motivations for these attitudes and the individual factors that best explained them. Using systematic sampling, a quantitative questionnaire was administered to 705 households at two sites in the Nepal Himalayas: Sagarmatha National Park, with a less decentralised governance model, and Annapurna Conservation Area, with a more decentralised model. Linear regression models were the main form of analysis. Based on these, attitudes to snow leopard conservation emerged as the strongest influence on local attitudes to snow leopards, and vice versa. This was true in both PAs, despite their differing management regimes. Other important explanatory factors included numbers of livestock owned, years of education, household livelihoods and age. Furthermore, a positive intrinsic motivation was the most common reason given by respondents to explain their attitudes to both snow leopards and snow leopard conservation. These findings demonstrate that, in addition to the usual suite of factors that influence attitudes to a species, the way in which its conservation is pursued and perceived also needs consideration. How the snow leopard is conserved may strongly influence its coexistence with local communities.

## Introduction

Coexistence with humans is a critical issue for all large carnivores [1], including the snow leopard *Panthera uncia*, a wild felid inhabiting mountainous areas of 12 South and Central Asian countries [2]. Snow leopards have recently been re-classed as Vulnerable on the IUCN Red List [3]. They face numerous anthropogenic threats, primarily associated with competition with people for habitat, prey and livestock [4–8], but also from climate change [9,10], economic developments such as infrastructure and mining [11], and illegal trade [12,13]. Approaches to snow leopard conservation have responded to these developing threats in various ways, such as involving local communities in conservation governance [14–16], and setting up conservation incentive and livestock insurance schemes [17–20].

Snow leopards occupy large home ranges which often lie fully or partially outside of protected areas (PAs) [16]. Therefore, their coexistence with human populations between and outside of PAs, and within PAs with human habitation, is key to the species’ persistence, as it is for many other large carnivore species [1,21,22], such as tigers *Panthera tigris* [23,24]. Human attitudes to snow leopards are likely to be an important factor in this process, but to date have only been considered comprehensively in India [25–28] and China [29,30]. An earlier study in the Annapurna Conservation Area (ACA), Nepal, found more than 60% of respondents were strongly negative towards snow leopards, but did not model explanatory factors [31]. Respondent characteristics from these more recent studies that positively influenced attitudes to snow leopards included: non-nativity [27]; diversified livelihoods [25,26]; male gender [26,28,29]; increased education levels [26,28]; lower ages [26]; increased religiosity [28]; increased livestock holdings [26]; and increased knowledge [28]. Such complexity has also been noted for attitudes to other species of carnivore, including felids [32–34]. However, a meta-analysis of factors driving attitudes towards large mammals found that predictors were not uniformly assessed across studies and that intangible costs was the most important predictor category overall [35]. Rationales for such attitudes included intrinsic, extrinsic, positive and negative reasons.

Attitudes to wildlife conservation, notably the actors and interventions involved, have been studied much less frequently than attitudes to wildlife itself. These lacunae may be, in part, because of conservation’s historical ontological bias towards natural, and apolitical, science [36]. Nevertheless, research and practice that considers attitudes to the social process that is conservation is a necessary part of human-wildlife coexistence. In particular, the potential for PAs to restrict as well as benefit local livelihoods makes it imperative to consider how they are perceived by inhabitants and neighbours [37–40]. A study in Ethiopia, for instance, found that positive attitudes to PA presence and PA staff were predicated upon benefits from the PA, as well as respondent age, family size and income source [38]. Elsewhere, links between attitudes to PAs and wildlife tourism have been found to be both positive in India [37] and negative in Indonesia [39], while increased knowledge has been associated with improved attitudes to Reserved Forests in India [41]. Gender can also shape attitudes towards wildlife conservation. A study in Nepal found no gender gap in attitudes to several PAs [42], yet women perceived more difficulties in resource extraction than men, a trend noted by others [43].

With the exception of a survey of local attitudes to a livestock compensation scheme in China [44], no studies to date have comprehensively considered attitudes towards snow leopard conservation in general, including gendered dimensions. While a study in eastern Nepal found mostly negative attitudes towards snow leopard conservation [45], its small sample size (n = 17) renders it unrepresentative. Rosen et al. [46] have called for research to address this critical knowledge gap, arguing that it ‘ …would highlight the dissonance between the meaning and significance of wildness to local societies and to outside conservationists. Nowhere is this disjuncture more prominent that in the debate over how to resolve the conflict between rural herders and snow leopards.’ Data on the reasons for attitudes to both snow leopards and snow leopard conservation are also lacking. Furthermore, understanding the potential links between attitudes to snow leopards and attitudes to their conservation is crucial for designing and implementing conservation programmes that garner support from local communities *and* successfully conserve snow leopards. While a likely correlation between these two variables may appear to be a valid assumption, it is still an assumption until it has been empirically tested. Therefore, this study aims to establish which factors best explain human attitudes to snow leopards and to snow leopard conservation, as well as the motivations for these attitudes. It did so by asking four questions:

1. What are individual respondents’ attitudes to snow leopards and why do they hold these views?
2. What are individual respondents’ attitudes to snow leopard conservation and why do they hold these views?
3. What factors best explain individual respondents’ attitudes to snow leopards?
4. What factors best explain individual respondents’ attitudes to snow leopard conservation?

## Methods

### Ethical approval

Ethical approval was provided by the Department of Geography’s Ethics Review Group at the University of Cambridge. Field research approval was provided by the Department of National Parks and Wildlife Conservation, Nepal, and by the National Trust for Nature Conservation, Nepal.

### Study areas

**Fig 1:**
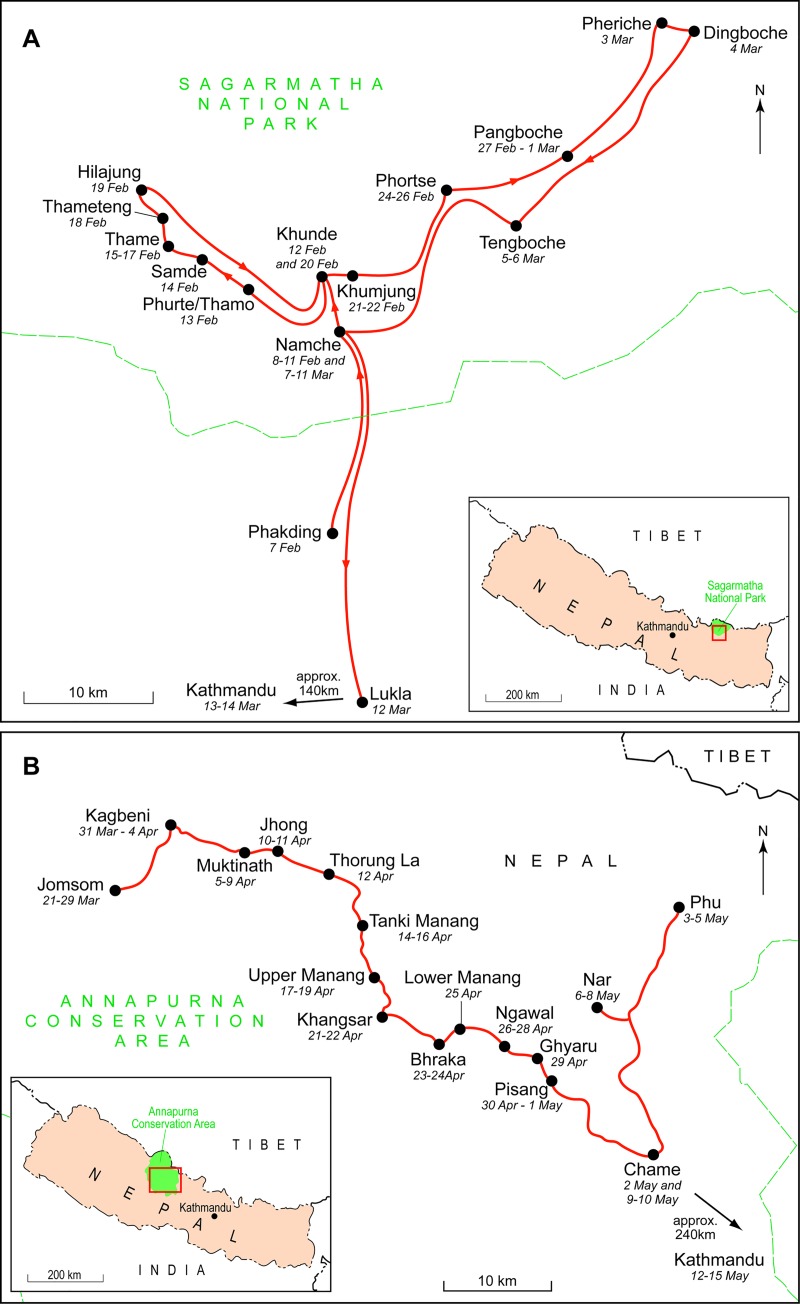
Study areas in Nepal showing areas and dates sampled. (A) Sagarmatha National Park. (B) Annapurna Conservation Area. Locations outside of study sites, and the dates visited, shown for illustrative purposes only.

Sagarmatha National Park (SNP; Fig 1) was established in the north-eastern part of Nepal in 1976 and a buffer zone was introduced in 2002 [47]. Habitat gradients exist between temperate oak and pine forests at 2,845m, to permanent snow at 8,848m [48]. Snow leopards prefer the sub-alpine, alpine and nival zones lying between 3,500 and 5,500m, and these support similar genera of vegetation to the parallel zones in ACA [49]. SNP has a population of Himalayan tahr *Hemitragus jemlahicus* [50] which are preyed upon by snow leopards, and over the last 15 years the species has recolonised the SNP after an absence of several decades following local extirpation [51,52]. In addition, there are also 3,500 people living in 63 settlements within SNP [48,53]. SNP is managed under a less decentralised conservation management regime, including with greater State involvement via the Department of National Parks and Wildlife Conservation. However, the amount of decentralised governance, in the form of local devolution, participation and revenue sharing, has been increasing since 2002 [54].

Gazetted in 1986, the Annapurna Conservation Area (ACA; Fig 1) comprises 7,629 km^2^ of protected landscape in north-central Nepal and is the country’s largest PA [47]. It comprises habitat gradients from sub-tropical sal forest at 790m to perennial snow at 8,091m. The sub-alpine, alpine and nival zones in ACA that snow leopards favour are characterised by alpine and steppe plant communities, including *Anenome*, *Hippophae*, *Gentiana*, *Salix*, *Carex*, *Myricaria*, *Kobresia and Populus* spp. [49]. The presence of blue sheep *Pseudois naur*, particularly in the Manang and Mustang districts, provides the primary prey species for a significant population of snow leopards [53–57]. An estimated 100,000 people also live within ACA [48], and participate in a decentralised conservation management regime with considerable degrees of local devolution, participation and revenue sharing. A co-management approach has been used, between local communities and a Nepali NGO, the National Trust for Nature Conservation [58]

### Questionnaire preparation

Data were mainly gathered through a household questionnaire with sections and questions that set out to address each of the four research questions concerning: (a) attitudes to snow leopards; (b) attitudes to snow leopard conservation; c) reasons for attitudes to snow leopards; (d) reasons for attitudes to snow leopard conservation (S1 File). In this case, the survey had both descriptive and confirmatory roles, profiling variables and testing relationships between them [59,60]. Nepali research assistants helped to extend the potential sampling scope. Following White [61], although the majority of the questionnaire was designed to involve closed questions measuring attitudes and other variables, additional open questions were added to assess respondents’ reasons for these attitudes. Furthermore, while this study considered the individual as the main unit of analysis, some factors were measured at the household level, and were included due to their relevance and the importance of multiscale factors in explaining attitudes [26,62].

Explanatory variables were included based on a review of the relevant literature and included a multidimensional livelihoods index based on the Sustainable Livelihoods Framework [63, 64, S1 Table]. In addition, a question on snow leopard identification utilised a photo plate of similar-sized carnivores present in one or both study sites, with typical species images taken from Wildscreen Arkive [65]. Unweighted summated rating scales were developed to measure local attitudes to snow leopards (S2 Table) and attitudes to snow leopard conservation, including both actors and interventions (S3 Table), following Spector [66]. These utilised five-point Likert scales, which, despite Suryawanshi et al’s [26] claim, can be used reliably in Asian cultures [67], including for snow leopard attitudinal assessment [31]. In this study the scales drew primarily upon Carter et al. [32, 68] for attitudes to snow leopards, and upon Nepal and Spiteri [69] for attitudes to snow leopard conservation. Variables included in the attitudes to snow leopard conservation scale were further refined by an in-depth review of the literature [2, 4, 14, 16, 18, 20, 25, 37, 38, 41, 42, 46] and by key focal interviews during the scoping phase of the study.

Before being used to collect data for the study, and after approval by the Ethics Committee of the Department of Geography at the University of Cambridge, the draft household questionnaire was trialled with a sample of 24 households in ACA. This allowed the draft indicators and questions to be assessed in relation to local conditions [70], drawing particularly on Newing et al.'s [71] checklist of practical tips for the pilot stage of a questionnaire-based research project. For logistical reasons, the trial stage ran parallel to the scoping phase, which also meant that the instrument could not be piloted in SNP as the relevant research permit was not ready. Appropriate modifications were then made to the survey instrument.

### Questionnaire administration

The data were collected from SNP and ACA between February and May 2014. The informal nature of many of the settlements and the absence of a sampling frame for them necessitated that systematic sampling was used instead. Census data provided the number of households in each Village Development Committee (renamed Gaunpalika from 2017) and 25% of these gave a target sample size of 700, which was considered a realistic and achievable goal given the time and resources available, as well as the snow leopard conservation literature reviewed [25–29]. In practice, and whether the settlement was clustered or linear, every fourth household in each settlement was sampled, using a similar approach to Paudel and Thapa [72], who sampled every third house at their study sites in the Middle Hills of Nepal. A specific concern here was the ease with which a household could be sampled more than once [71], especially with two research assistants at work alongside the principal investigator and a fieldwork manager. To prevent any such overlap in area or in sampling, on entering a settlement, the research assistants made a plan to divide the settlement into two halves, with each having responsibility for data collection in one half of the settlement.

In addition, back-checking of a proportion of surveys carried out by research assistants was implemented to promote and ensure reliability [61,71]. A random number generator was used to select approximately 10% of households to back-check. Once respondents were located they were asked to confirm when they had completed the survey, how long it had taken and to describe the research assistant who had conducted it. The back-checking process uncovered no cases of fraudulent data collection.

### Questionnaire analysis

Multi-variable scales were developed and used in this study. When amalgamated from their respective categorical variables, these were all treated as continuous data for the purposes of analysis. This follows the recent trends in snow leopard [26,29], tiger [32] and jaguar [73] attitudinal analyses, which have used regression models to explain rather than to predict [74]. Because Likert scales were used, where a lower number equalled a more positive attitude, the final attitudinal scales were reverse-scored [69]. This allowed for positive correlations to be more clearly displayed and communicated. Binary respondent variables–gender, nativity, religion and religiosity–were all scored as either 0 or 1 to enable means for each to be calculated and compared between sites. Due to significant variation, data for household livestock owned and household livestock lost to snow leopards were changed to a log^10^ scale before inferential analysis, following Zimmermann et al. [75].

To test for reliability, independent t-tests were used to test for inter-observer consistency between the two research assistants, rather than Levene's test for homogeneity of variance, as a large sample can skew its results [76]. In addition, and after coding the responses to the open questions, inter-coder reliability was then tested using paired sample t-tests, and no questions were found to have been inconsistently coded [77]. Internal reliability was then tested for the two composite scales by using Cronbach’s alpha. The score for the snow leopard attitudinal scale was 0.878, while the score for the snow leopard conservation attitudinal scale was 0.664. While some authors have argued on theoretical grounds that only scores of >0.7 are reliable [78], in practice, test scores of >0.6 are reported in the medical [79,80] and conservation literature [69]. Given that only one of the scales in this study has a test score of <0.7, and that this example is at the upper end of 0.6–0.7 range, internal reliability of the scales was considered to be acceptable.

Descriptive and inferential statistics were used to analyse the data. Independent t-tests were used to compare respondent and attitudinal results between SNP and ACA. For both sites and jointly, multiple regression models were used to test the variables explaining attitudes to snow leopards and to snow leopard conservation. Prior to constructing the models, the data were checked to ascertain whether they met the necessary assumptions: linearity, reliability, homoscedasticity and normality [81]. Furthermore, multicollinearity between variables was assessed and none were found to exceed the recommended limit of 0.7 [76,82]. Hierarchical entry based on theoretical suitability was used, as entry based on statistical significance alone can be biased by the number of predictors and subject to severe artefacts [76,83]. Model selection to determine the most suitable, parsimonious model used the r^2^ change results to determine goodness-of-fit [83]. P-P plots to test for normality in multiple regression models indicated some evidence of non-normality, and bootstrapping was therefore used for all models [76]. Qualitative data concerning respondents’ motivations for their attitudes was analysed with descriptive statistics following coding and categorisation.

## Results

### Respondent attributes

Of the joint sample of 705, 260 (36.9%) were from SNP and 445 (63.1%) were from ACA. The mean age of respondents was 42.7 years, and ranged between 16 and 86 years of age, respectively. There was no significant difference in age between SNP and ACA (*t* = -1.83, *p* = 0.067). This was also the case for years of education (*t* = 0.89, *p* = 0.38), which varied between 0 and 18, with a mean of 3.9 years of age. Males comprised 52.1% of those surveyed, and females 47.9%, with the SNP sample tending slightly towards more males than in ACA (*t* = 1.98, *p* = 0.048). Most respondents were native (89.4%) rather than non-native (10.6%). However, there were significantly more non-locals in SNP than in ACA (*t* = -3.58, *p* = 0.001). Although five categories of religion were used, the low scores in all except Buddhist (91.6%) meant that the other four categories–none, Bon, Hindu and other—were aggregated into a single score (8.4%). ACA was significantly more Buddhist than SNP (*t* = 2.92, *p* = 0.004). Likewise, the five-point Likert scale used to measure religiosity was collapsed into two categories due to a lack of data for categories three to five. Very religious respondents comprised 59.1% of those surveyed while less than very religious equalled 40.1%, with the ACA sample being significantly more religious than the SNP sample (*t* = -3.45, *p* = 0.001). Finally, 53.3% of respondents were able to positively identify a snow leopard, compared to 46.7% who were not. The identification rate was significantly higher in ACA than in SNP (*t* = 4.71, *p* = 0.001).

A number of respondent attributes were also considered at the household level. Household Sustainable Livelihoods Index scores varied between 0.21 and 0.77, with a mean of 0.55. They were significantly higher in ACA than in SNP (*t* = -2.42, *p* = 0.016). The same was true of livestock owned per household (*t* = 8.74, *p* = 0.001), with an overall mean for the combined sample of 15.48, with a range between 0 and 250. Accordingly, self-reported livestock losses to snow leopards in the previous 12 months in ACA were also significantly higher than in SNP (*t* = -2.42, *p* = 0.001). Mean losses per household lay between a minimum of 0 and a maximum of 21. Fourthly, mean household size (3.81) lay in the range of 0 and 12 in the joint sample but household size was significantly higher in ACA than in SNP (*t* = 3.17, *p* = 0.002).

### Attitudes to snow leopards

Overall, 10.4% of respondents were very positive towards snow leopards, while 50.1% were positive. Meanwhile, 19.0% were neutral, 16.5% were negative and 4.1% were very negative. Among the joint sample’s future preference for snow leopards in their area, 16.5% were very positive, 44.6% were positive, 15.2% were neutral, 17.4% were negative and 6.1% were very negative. Mean results of both of these variables, as well as of the snow leopard attitudinal scale into which they were aggregated, were computed (Table 1).

**Table 1:** Mean attitudes to snow leopards in the combined sample from Sagarmatha National Park (SNP) and Annapurna Conservation Area (ACA), also showing differences in attitudes between sites.

	Combined N = 705	SNP N = 260	ACA N = 445	Difference between PAs
Attitudes to snow leopards	2.54 (1.02)	2.44 (0.99)	2.60 (1.03)	*t* (703) = -2.01*
Preference for future presence of snow leopards	2.52 (1.14)	2.43 (1.09)	2.57 (1.16)	*t* (703) = -1.65*
**Snow leopards attitudinal scale**	**2.53 (1.02)**	**2.43 (0.97)**	**2.59 (1.04)**	*t* (703) = -1.93*
**Reverse scored snow leopard attitudinal scale**	**3.47 (1.02)**	**3.57 (0.97)**	**3.42 (1.04)**	*t* (703) = 1.93*

Standard deviations in parentheses and independent t-test of differences between study sites. Based on Likert scales ranging from (1) very positive to (5) very negative. Cronbach's alpha for snow leopard attitudinal scale = 0.878.

* *p* = >0.05.

A wide range of reasons were given by respondents for their attitudes towards snow leopards (Table 2). Positive intrinsic motivations, such as cultural and religious factors related to the inherent value of the species, were the most common. Negative reasons, related to the challenges of coexisting with a predatory species and its real or perceived danger to livestock, were the next most frequently listed. Positive extrinsic factors came third in this table. These refered to the real or perceived benefits or uses that locals felt may accrue from snow leopards, such as controlling wild herbivore populations or attracting tourists.

**Table 2:** Reasons for attitudes to snow leopards for a combined sample, in Sagarmatha National Park (SNP) and Annapurna Conservation Area (ACA).

Scale item	Sample	N	Reason(s) (%)					
			None	Positive intrinsic	Positive extrinsic	Multiple positive	Positive and negative	Negative
**Snow leopards**	Joint	705	11.5	37.6	15.5	9.3	4.4	21.6
	SNP	260	8.1	32.9	19.8	13.2	4.7	21.3
	ACA	444	13.6	40.4	12.9	6.9	4.3	21.8
**Future presence of snow leopards**	Joint	676	12.5	37.9	9.4	8.1	8.7	23.4
	SNP	258	11.2	28.8	15.0	12.3	6.5	26.2
	ACA	418	13.3	37.4	6.1	5.6	9.9	27.7
**Overall snow leopards**	**Joint**	**1381**	**12.0**	**37.8**	**12.4**	**8.7**	**6.6**	**22.5**
	**SNP**	**518**	**9.6**	**30.9**	**17.4**	**12.8**	**5.5**	**23.8**
	**ACA**	**862**	**13.5**	**38.9**	**9.5**	**6.2**	**7.1**	**24.8**

**Fig 2:**
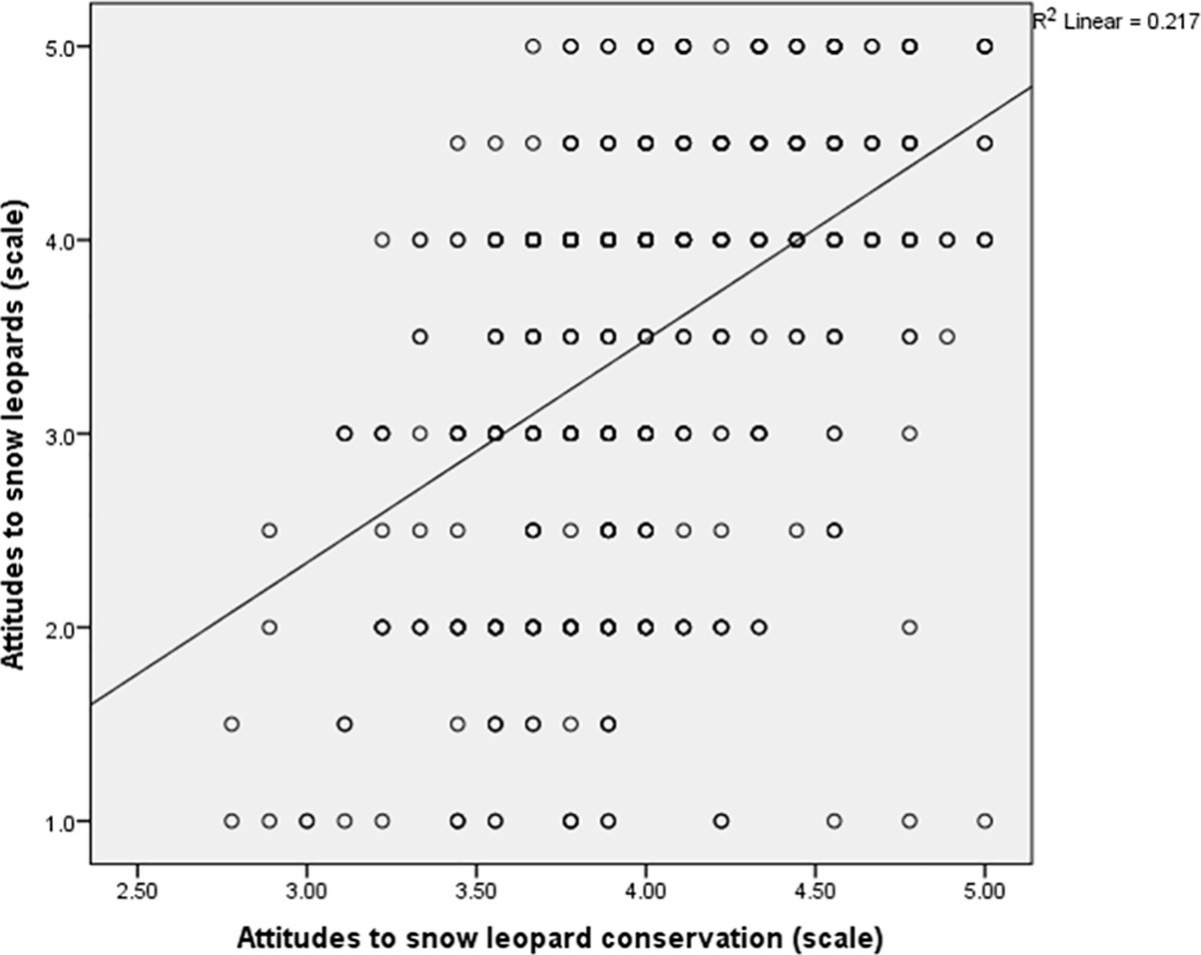
Scatterplot showing a strong positive relationship between attitudes to snow leopard conservation and attitudes to snow leopards. N = 678. Scales comprised means from reverse-scored five-point Likert scale questions. Snow leopard attitudinal scale components: snow leopards; future presence of snow leopards; Cronbach’s alpha = 0.878. Snow leopard conservation attitudinal scale components comprise: park management; local conservation committee; snow leopard killing ban; snow leopard prey killing ban; livestock compensation scheme; corral construction; environmental education; non-timber forest product collection limits; wood collection limits; Cronbach’s alpha = 0.664.

A number of key respondent attributes and household characteristics best explained attitudes to snow leopards (Table 3). For both PAs, and for a combined sample, respondents’ attitudes to snow leopard conservation was the most important factor by a large margin (Fig 2). Several of the non-significant variables in the final joint regression model showed significant relationships with attitudes to snow leopard in bivariate analysis. Respondents who had higher household Sustainable Livelihoods Index scores (*B* = 2.03, R^2^ = 0.049, *p* = 0.001), who were younger (*B* = -0.020, R^2^ = 0.088, *p* = 0.001), who were non-native (*t* = 3.38, *p* = 0.005), who could positively identify a snow leopard (*t* = -2.26, *p* = 0.027), who were non-Buddhist (*t* = -4.14, *p* = 0.002) and who were less than very religious (*t* = 3.89, *p* = 0.001) were significantly more positive towards snow leopards.

**Table 3:** Significant variables explaining attitudes to snow leopards in Sagarmatha National Park, in Annapurna Conservation Area and for a combined sample.

Combined		Sagarmatha National Park		Annapurna Conservation Area	
N = 608	R^2^ = 0.39	N = 238	R^2^ = 0.42	N = 370	R^2^ = 0.38
Explanatory factor	1 *b*	Explanatory factor	1 *b*	Explanatory factor		1 *b*
	*2 SE B*		*2 SE B*			*2 SE B*
	3 Standardised *b*		3 Standardised *b*			3 Standardised *b*
Attitudes to snow leopard conservation	1 0.96 (0.76,1.15) 2 0.096 3 0.38*	Attitudes to snow leopard conservation	1 0.78 (0.76,1.15) 2 0.17 3 0.32*	Attitudes to snow leopard conservation	1 1.07 (0.81, 1.31) 2 0.13 3 0.41*
Number of household livestock owned (log^10^ scale)	1–0.29 (-0.43, -0.15) 2 0.070 3–0.17*	Number of household livestock owned (log^10^ scale)	1–0.46 (-.071,-0.22) 2 0.13 3–0.23*	Years of education	1 0.047 (0.022,0.072) 2 0.013 3 0.21*
Years of education	1 0.15 (0.015,0.053) 2 0.009 3 0.15*	Gender~	1–0.36 (-0.57,-0.14) 2 0.12 3 -.018*	Number of household livestock killed by snow leopards (log^10^ scale)	1–0.64 (-0.99,-0.30) 2 0.18 3–0.17*
Number of ousehold livestock killed by snow leopards (log^10^ scale)	1–0.66 (-0.43,-0.15) 2 0.17 3–0.15*	Household Sustainable Livelihoods Index score	1 0.99 (0.12,1.88) 2 0.47 3 0.12**	Number of household livestock owned (log^10^ scale)	1–0.22 (-0.41,-0.041) 2 0.090 3–0.13**
Gender~	1–0.26 (-0.40, -0.12) 2 0.075 3–0.13*	Number of household livestock killed by snow leopards (log^10^ scale)	1–0.99 (-1.93,-0.096) 2 0.46 3–0.12**	Gender~	1–0.25 (-0.45,-0.070) 2 0.097 3–0.12*
Constant	1–0.22 (-1.03, 0.64) 2 0.44 *p* = 0.63	Constant	1 0.86 (-0.52, 2.29) 2 0.69 *p* = 0.20	Constant	1 0-.70 (-1.86, 0.73) 2 0.64 *p* = 0.27

Linear models of factors explaining individual attitudes to snow leopards, with 95% bias corrected and accelerated confidence intervals reported in parentheses. Confidence intervals and standard errors were based on 1000 bootstrap samples. Rankings based on standardised *b* scores.

* *p* = ≤0.01.

** *p* = ≤0.05.

~ 0 = male; 1 = female.

### Attitudes to snow leopard conservation

We first considered attitudes to snow leopards among conservation actors. For the joint sample, 18.9% of respondents were very positive towards park management, 47.2% were positive, 27.0% were neutral, 5.3% were negative and 1.6% were very negative. For local conservation committees, 15.2% of the overall sample were very positive, 39.8% were positive, 43.3% were neutral, 1.5% were negative and 0.3% were very negative. The mean results of both of these variables were calculated, as was the snow leopard conservation attitudinal scale into which they were aggregated (Table 4). Attitudes to snow leopard conservation actors were significantly lower in ACA, although there was no difference in overall conservation attitudes between SNP and ACA.

**Table 4:** Mean attitudes to snow leopard conservation in the combined sample from Sagarmatha National Park (SNP) and Annapurna Conservation Area (ACA), also showing differences in attitudes between sites.

Attitudes towards	N	Joint	N	SNP	N	ACA	Difference
Park management	703	2.23 (0.87)	260	1.93 (0.75)	443	2.41 (0.89)	*t* (620) = -7.56*
Local conservation committee(s)	679	2.32 (0.75)	260	2.13 (0.76)	419	2.44 (0.73)	*t* (677) = -5.24*
**Conservation actors combined**	**679**	**2.29 (0.65)**	**260**	**2.03 (0.63)**	**419**	**2.43 (0.81)**	*t* (677) = -8.27*
Ban on the killing of snow leopards	703	1.85 (0.89)	260	1.70 (0.72)	443	1.94 (0.96)	*t* (663) = -3.84*
Ban on the killing of snow leopard prey	702	1.63 (0.62)	260	1.61 (0.61)	442	1.64 (0.63)	*t* (700) = -0.59
Livestock compensation scheme	701	2.23 (0.86)	260	2.41 (0.89)	441	2.12 (0.83)	*t* (516) = 4.27*
Corral construction	701	2.07 (0.78)	260	2.59 (0.64)	441	1.77 (0.68)	*t* (699) = 15.63*
Environmental education	701	1.80 (0.71)	260	1.73 (0.64)	441	1.83 (0.74)	*t* (608) = -1.83
Non-Timber Forest Product collection limits	701	2.08 (0.84)	260	2.32 (0.82)	441	1.95 (0.82)	*t* (541) = 5.78*
Wood collection limits	701	1.76 (0.77)	260	1.72 (0.87)	445	1.79 (0.71)	*t* (462) = -1.10
**Conservation interventions combined**	**701**	**1.92 (0.46)**	**260**	**2.01 (0.42)**	**445**	**1.86 (0.48)**	*t* (601) = 4.29*
**Snow leopard conservation attitudinal scale**	**678**	**2.00 (0.41)**	**260**	**2.00 (0.39)**	**418**	**2.00 (0.42)**	***t* (676) = -0.012**
**Reverse scored snow leopard conservation attitudinal scale**	**678**	**3.98 (0.41)**	**260**	**3.98 (0.39)**	**418**	**3.98 (0.42)**	***t* (676) = 0.012**

Mean snow leopard conservation attitudes results in SNP, ACA and combined, with SD in parentheses and independent t-test of differences between study sites. Based on Likert scales ranging from (1) very positive to (5) very negative. Cronbach's alpha for snow leopard conservation attitudinal scale = 0.664.

* *p* = ≤0.05.

Attitudes towards snow leopard conservation interventions were generally more positive than towards organisations involved in snow leopard conservation (Table 4). We summarise key results in the following section. Towards the ban on the killing of snow leopards, 39.5% of the overall sample was very positive and 43.1% was positive Respondents held a similar view of the ban on the killing of snow leopard prey: 43.4% were very positive and 51.4% were positive. However, the livestock compensation scheme was supported by only 19.4% of overall respondents who were very positive towards it, while 44.9% were positive and 31.1% were neutral. For corral construction, 25.7% of the joint sample was very positive, 42.2% was positive and 31.1% was neutral. Overall, 37.4% of those surveyed were very positive towards environmental education activities, while 45.6% were positive. For attitudes to collection limits on Non-Timber Forest Products, though, there was less support for the combined sample. Twenty-six percent were very positive, 44.5% were positive and 25.2% were neutral. Lastly, towards wood collection limits, 39.9% of the joint sample were very positive and 47.5% were positive.

Respondents reported various factors that motivated their attitudes to snow leopard conservation (Table 5). As with attitudes to snow leopards, positive intrinsic reasons were the most common motivation. These reflected a range of social, cultural and religious influences relating to the inherent value of conservation. In stark contrast to attitudes to snow leopards, positive extrinsic factors were much more important for attitudes to the conservation of the species. These referred to a range of real and perceived benefits that accrue to local communities from snow leopard conservation. Negative motivations for attitudes to the various actors and interventions that make up this process were much less frequent than for attitudes to the species itself.

**Table 5:** Factors best explaining attitudes to snow leopard conservation for a combined sample, in Sagarmatha National Park (SNP) and Annapurna Conservation Area (ACA).

Scale item	Site	N	Reason(s) (%)					
			None	Positive intrinsic	Positive extrinsic	Multiple Positive	Positive and negative	Negative
**Park management**	Joint	701	19.1	40.9	13.0	12.7	3.9	10.4
	SNP	259	10.0	65.6	6.2	12.0	3.1	3.1
	ACA	442	24.4	26.5	17.0	13.1	4.3	14.7
**Local conservation committee**	Joint	673	40.6	25.4	17.8	12.5	0.4	3.3
	SNP	259	29.3	44.4	5.4	18.5	1.2	1.2
	ACA	414	47.6	13.5	25.6	8.7	0.0	4.6
**Overall conservation actors**	**Joint**	**1374**	**29.9**	**33.1**	**15.4**	**12.6**	**2.1**	**6.9**
	**SNP**	**518**	**19.7**	**55.0**	**5.8**	**15.3**	**2.1**	**2.1**
	**ACA**	**856**	**36.0**	**20.0**	**21.3**	**10.9**	**2.1**	**9.7**
**Snow leopard killing ban**	Joint	701	7.6	63.3	4.9	13.0	4.1	7.1
	SNP	259	6.6	61.8	5.8	21.6	2.3	1.9
	ACA	442	8.1	64.3	4.3	7.9	5.2	10.2
**Snow leopard prey killing ban**	Joint	695	1.7	77.3	2.9	16.3	0.3	1.6
	SNP	259	1.9	66.4	6.2	23.9	0.8	0.8
	ACA	436	1.6	83.7	0.9	11.7	0.0	2.1
**Livestock compensation scheme**	Joint	699	12.9	1.3	38.6	1.8	24.4	21.0
	SNP	260	18.5	1.2	27.7	2.3	24.6	25.8
	ACA	439	9.7	1.3	44.9	1.6	24.3	18.2
**Corral construction**	Joint	668	18.3	0.0	67.4	1.8	0.6	11.9
	SNP	235	33.5	4.6	36.2	0.0	0.8	25.0
	ACA	433	9.4	0.0	85.6	0.2	0.4	4.3
**Environmental education activities**	Joint	700	15.0	54.4	10.6	18.0	0.0	2.0
	SNP	260	8.1	51.2	7.3	31.9	0.0	1.5
	ACA	440	19.1	56.4	12.5	9.8	0.0	2.3
**Non-Timber Forest Product collection limit**	Joint	698	12.3	23.1	40.7	5.0	1.4	17.5
	SNP	258	16.3	18.2	25.6	7.8	2.7	29.5
	ACA	440	10.0	25.9	49.5	3.4	0.7	10.5
**Wood collection limits**	Joint	700	3.6	31.9	51.1	4.7	3.6	5.1
	SNP	259	1.9	36.3	41.7	5.4	6.9	7.7
	ACA	441	4.5	29.3	56.7	4.3	1.6	3.6
**Overall conservation interventions**	**Joint**	**4861**	**10.2**	**35.9**	**30.9**	**8.6**	**4.9**	**9.5**
	**SNP**	**1790**	**12.3**	**34.2**	**21.4**	**13.3**	**5.4**	**13.4**
	**ACA**	**3071**	**8.9**	**37.3**	**36.3**	**5.6**	**4.6**	**7.3**
**Overall snow leopard conservation**	**Joint**	**6235**	**14.5**	**35.3**	**27.5**	**9.5**	**4.3**	**8.9**
	**SNP**	**2308**	**13.9**	**38.8**	**18.0**	**13.7**	**4.7**	**10.9**
	**ACA**	**3927**	**14.9**	**33.4**	**33.0**	**6.8**	**4.1**	**7.8**

Various respondent attributes and household characteristics best explained attitudes to snow leopard conservation (Table 6). Attitudes to snow leopards was consistently the most important factor across both sites and overall. Of the non-significant variables in the final joint regression model, a number had significant relationships with attitudes to snow leopard conservation in bivariate analysis. Respondents who had more years of education (*B* = 0.023, R^2^ = 0.062, *p* = 0.001), who had fewer household livestock killed by snow leopards (*B* = -0.021, R^2^ = 0.013, *p* = 0.008) and who were non-native (*t* = 2.32, *p* = 0.021) were significantly more positive towards snow leopard conservation. The remaining variables did not have significant relationships with the dependent variable.

**Table 6:** Factors best explaining attitudes to snow leopard conservation in Sagarmatha National Park, in Annapurna Conservation Area and for a combined sample.

Combined		Sagarmatha National Park		Annapurna Conservation Area	
N = 585	R^2^ = 0.59	N = 237	R^2^ = 0.56	N = 346	R^2^ = 0.54
Explanatory factor	1 *b*	Explanatory factor	1 *b*	Explanatory factor	1 *b*
	*2 SE B*		*2 SE B*		*2 SE B*
	3 Standardised *b*		3 Standardised *b*		3 Standardised *b*
Attitudes to snow leopards	1 0.16 (0.13,0.20) 2 0.018 3 0.41*	Attitudes to snow leopards	1 0.15 (0.081, 0.20) 2 0.031 3 0.36*	Attitudes to snow leopards	1 0.18 (0.13, 0.22) 2 0.023 3 0.46*
Household Sustainable Livelihoods Index score	1 0.47 (0.19, 0.78) 2 0.14 3 0.13*	Household Sustainable Livelihoods Index score	1 0.79 (0.44, 1.13) 2 0.19 3 0.24*	Native~	1–0.27 (-.045, -0.094) 2 0.090 3–0.17*
Age	1–0.003 (-0.001, -0.005) 2 0.001 3–0.11*			Age	1–0.004 (-.0006, -0.001) 2 0.001 3–0.14*
Positive identification of snow leopard~	1 0.083 (0.26, 0.14) 2 0.029 3 0.10*			Positive identification of snow leopard~	1 0.089 (0.19, 0.16) 2 0.036 3 0.11*
Constant	1 3.32 (3.11, 3.53) 2 0.11 *p* = 0.001	Constant	1 3.16 (2.83, 3.50) 2 0.16 *p* = 0.001	Constant	1 .3.61 (3.30, 3.93) 2 0.15 *p* = 0.001

Linear models of factors explaining individual attitudes to snow leopard conservation, with 95% bias corrected and accelerated confidence intervals reported in parentheses. Confidence intervals and standard errors based on 1000 bootstrap samples. Rankings based on standardised *b* scores.

* *p* = ≤0.01.

** *p* = ≤0.05.

~ 0 = no; 1 = yes.

## Discussion

### Attitudes to snow leopards

Local attitudes to snow leopards have greatly improved since the first study was concluded in ACA in 1993 [31]. Indeed, the percentage of respondents identifying as very negative towards snow leopards has fallen from over 60% to 4% in the two decades since [31]. This may be due, in part, to conservation efforts in the area [84]. Unfortunately, attitudes to the species in SNP had not been assessed previously and so a comparison could not be made. The fact that attitudes were the same across both PAs demonstrates that factors other than governance models may be influencing them most strongly. In addition, the increasing decentralisation of conservation governance in SNP since 2002 has reduced the previously marked differences between management approaches in ACA and SNP. Elsewhere, attitudes to snow leopards have varied considerably, from more negative in parts of India [25,27], to less negative in parts of China and in other parts of India [26,28–30]. The link between attitudes in the present and preferences for future presence of a species has been observed with tigers in Nepal [32], where it was found to be strongly linked, but has not been assessed for snow leopards elsewhere.

As is the case with questionnaire research in conservation and ecology generally, respondents’ motivations for their attitudes have been considered infrequently [61]. In this study, we have sought to understand the reasons for individuals’ attitudes to snow leopard and their conservation. The significance of positive intrinsic reasons for respondents’ attitudes to snow leopards would seem to corroborate previous research on the importance of socio-cultural factors as motivations for attitudes to snow leopards [85] and to other species of large carnivore [33–35].

This study considered attitudes to snow leopard conservation as a potential explanatory variable for wider attitudes towards snow leopards. Indeed, this was the most significant factor at both sites, despite their differing degrees of conservation governance decentralisation, including the greater level of community involvement in snow leopard conservation in ACA, historically and currently. Despite the lack of empirical precedent, this result confirms the significance of considering how the methods used to conserve a species may impact on attitudes to the species itself [46]. Other significant factors in the model have been found to be significant for explaining more positive attitudes to snow leopard elsewhere, including increased livestock ownership and loss [26], male gender [26,28,29], increased education [26,28] and diversified livelihoods [25,26].

Other variables that were significant during bivariate analysis here, but not in the study’s multivariate models, have also been found to be significant in other analyses. Non-nativity [27], increased knowledge [28] and lower ages [26] were all associated with more positive attitudes here and elsewhere. Increased religiosity was found to be positively linked with positivity to snow leopards amongst Buddhists in North-West India [28]. In contrast, religiosity was negatively correlated in this study. In addition, we found that non-Buddhists were more positive to snow leopards than were Buddhists, a pattern that was also observed in the same Indian study [28]. Clearly, when all relevant demographic factors are taken into account, religion and religiosity appear to be less important than previously thought. Yet the factors explaining attitudes to snow leopards remain multi-faceted and complex.

### Attitudes to snow leopard conservation

A similar complexity is also true for attitudes to the conservation of snow leopards. Attitudes to the various actors and interventions included in the study ranged from the ban on the killing of snow leopard prey, the most popular, to local conservation committees, the least popular. Although there was no difference in overall conservation attitudes between SNP and ACA, attitudes to snow leopard conservation actors were significantly lower in ACA. This is seemingly at odds with the suggestions in the general [86–88] and snow leopard conservation literature [16,89] that decentralised management should improve attitudes to conservation. In reality, it may be due to the increased presence and accessibility of conservation actors in ACA. Research from India, where it was found that participation in forest management groups was correlated with negative attitudes to Reserved Forests [41], would support our findings.

In contrast to attitudes to conservation actors, it is the sample from ACA that is significantly more positive to snow leopard conservation interventions than the sample from SNP. This seems to confirm the findings of a study from several PAs in South Asia, including ACA, where respondents were more positive about PA presence in general but less positive about PA staff in particular [90]. This appears to be the case for snow leopard conservation in ACA, while the reverse is true in SNP. In terms of reasons for these attitudes, we found equal results in the positive intrinsic and extrinsic valuation categories. In turn, this suggests that people value snow leopard conservation for how it can benefit them, as well as benefit the snow leopard. Both the snow leopard [16,46] and general [91,92] conservation literature have suggested that meeting the needs of humans as well as wildlife is important for conservation to establish and maintain popularity amongst local communities.

Multivariate analyses of attitudes to snow leopard conservation have not been studied previously, making it difficult to compare explanatory factors with other range states. However, because attitudes to snow leopards were found to be the most important variables in models for both SNP and ACA, it reiterates the relevance of the link between attitudes to the species and to its conservation, as has been briefly noted before in China [44] and Nepal [45]. For the other significant variables in the models, significant positive relationships with attitudes to conservation have been noted for livelihood diversification [38] and knowledge [41]. However, the positive correlation with age noted here was found to be a negative association in Ethiopia [38], while nativity was missing as a factor altogether in these other analyses.

A number of other variables that were not significant in the multivariate models were found to be significant during bivariate analysis, and have been found to be significantly linked to conservation attitudes elsewhere, including education [38,90] and household livestock killed [38]. The remaining explanatory variables did not show any significant relationship with attitudes to snow leopard conservation. With gender, this corroborates a study that found no gender gap with attitudes to several PAs in Nepal [42]. With household size, this study found the opposite of one in Ethiopia, where larger family sizes were correlated with more support for PAs [38]. Thirdly, the lack of a significant relationship between religion and attitudes or religiosity and attitudes, suggests that these factors are less important in explaining perceptions of snow leopard conservation than previously thought, as with attitudes to snow leopards [28].

## Conclusions

This research adds to a growing body of knowledge on attitudes to snow leopards [25,26,28–31], as well as on attitudes to other large felids and carnivores [32,33,73,75] and other large mammals [35]. As these studies demonstrate, the factors that best explain these attitudes vary across sites, although a few, such as gender, appear more frequently than others. Crucially, this study also added attitudes to snow leopard conservation to the explanatory model, and found that it was the most significant variable across both study sites. Along with parallel analyses of attitudes to snow leopard conservation, including explanatory regression models, this research represents the first known comprehensive empirical analysis of the factors that motivate and explain attitudes to the conservation of this species. In doing so, it addresses an important information gap highlighted by Rosen et al. [46], and complements exploratory work on attitudes to aspects of snow leopard conservation conducted elsewhere in Nepal [45] and in China [44].

Future research could replicate this study at other sites in snow leopard habitat where similar interventions and actors exist. Future research could also focus on replicating this research with other large felid, carnivore and mammal species to clarify potential linkages between attitudes to species and attitudes to the methods employed to conserve them. That attitudes to snow leopard conservation best explain attitudes to snow leopards underlines the importance of considering how wildlife conservation is perceived and pursued. How the snow leopard, and other species, are conserved may strongly influence their future coexistence with humanity.

## Supporting information

S1 File Study Questionnaire. (PDF)

S1 Table Sustainable Livelihoods Index variables and weighting. (DOCX)

S2 Table Snow leopard attitudinal scale variables and weighting. (DOCX)

S3 Table Snow leopard conservation attitudinal scale variables and weighting. (DOCX)
