# Supplementary material for: What factors best explain attitudes to snow leopards in the Nepal Himalayas?
Source: PLoS One. 2019 Oct 23;14(10):e0223565. doi: 10.1371/journal.pone.0223565 (PMC6808326; doi:10.1371/journal.pone.0223565)
Supplement: S1 File — (PDF) [file pone.0223565.s001.pdf]

Research Assistant initials ___

Human dimensions of snow leopard conservation: Household Questionnaire

Section 1 Household

1.1 Household location

1.1.1 VDC

1.1.2 Settlement

1.1.3 Head of household's name.

.
1.1.4 House name/distinguishing features

1.1.5 Adjacent landmark(s)

1.2 Human assets

1.2.1 How many household members are there?

Total	Adults (18+)	School age children (4-18)	Infants (<4)

1.2.2 How many adult household members can read and write?

	N/A	

1.2.3 How many household members of school age are in education?

	N/A	

Which of the following types of medical treatment does your household have access to?

		Yes	No	N/A
1.2.4	Self-administered traditional medicine			
1.2.5	Self-administered 'Western' medicine			
1.2.6	Visit to local clinic in PA			
1.2.7	Visit to clinic outside of PA			

Which of the following types of media does your household have access to?

		Yes	No	N/A
1.2.8	Newspaper			

1.2.9	Radio			
1.2.10	Television			
1.2.11	Internet			

1.3 Natural assets

1.3.1 Does your household have access to land for grazing animals?

Yes		No		N/A	

How many of each type of livestock does your household own?

	Livestock	Number	N/A
1.3.2	Cattle		
1.3.3	Sheep/goats		
1.3.4	Horses/mules/donkeys		
1.3.5	Yaks/yak hybrids		
1.3.6	Other		

1.3.7 Does your household have access to land for agriculture/cultivation?

Yes		No		N/A	

1.3.8 Are you able to sell any surplus agricultural products from your land?

Yes		No		N/A	

Which of the following natural products does your household you have access to?

		Yes	No	N/A
1.3.9	Fuelwood			
1.3.10	Construction wood			
1.3.11	Human food			
1.3.12	Animal food			
1.3.13	Medicinal plants			

Which of the following forms of water supply does your household have access to?

		Yes	No	N/A
1.3.14	Spring			
1.3.15	Well			
1.3.16	Handpump			
1.3.17	Outside tap			
1.3.18	Inside tap			

1.4 Social assets

Is anyone in your household a member of any of the following formal groups/organisations?

		Yes	No	N/A
1.4.1	Conservation committee			
1.4.2	Village development committee			
1.4.3	Tourism association			
1.4.4	Microcredit group			
1.4.5	Co-operative			
1.4.6	Women’s group			

1.4.7	School association			
1.4.8	Youth group			
1.4.9	Other			

Does your household have access to political representatives at the following levels?

		Yes	No	N/A
1.4.10	Local			
1.4.11	District			
1.4.12	National			

1.5 Physical assets

Which of the following types of fuel sources does your household have access to?

		Yes	No	N/A
1.5.1	Fuelwood			
1.5.2	Cylinder gas			
1.5.3	Kerosene oil			
1.5.4	Electricity			
1.5.5	Animal dung			
1.5.6	Other			

Which of the following types of buildings does your household have access to?

		Yes	No	N/A
1.5.7	Residential building			
1.5.8	Joint tourist/residential building			
1.5.9	Tourist building			
1.5.10	Other building			

Which of the following forms of transport does your household have access to?

		Yes	No	N/A
1.5.11	Foot			
1.5.12	Animal			
1.5.13	Bicycle			
1.5.14	Bus/taxi			
1.5.15	Aeroplane			
1.5.16	Motorcycle			
1.5.17	Other			

1.6 Financial assets

1.6.1 What was your total household income in the last 12 months (NR)?

0– 50,000	50,001–100,000	100,001–150,000	150,000-200,000	200,001-250,00	>250,001	Prefer not2say	N/A

Which of the following types of financial income does your household have access to?

		Yes	No	N/A
1.6.2	Livestock			
1.6.3	Agriculture/cultivation			
1.6.4	Wood			

1.6.5	Other natural products			
1.6.6	Tourism			
1.6.7	Remittances			
1.6.8	Savings			
1.6.9	Loans			
1.6.10	Other			

What are your most important sources of financial income as a household? RANK WITH 1 BEING THE MOST IMPORTANT.

		Rank
1.6.11	Livestock	
1.6.12	Agriculture/cultivation	
1.6.13	Wood	
1.6.14	Other natural products	
1.6.15	Tourism	
1.6.16	Remittances	
1.6.17	Savings	
1.6.18	Loans	
1.6.19	Other	

Section 2 Household conflict

2.1 Conflict with snow leopards

2.1.1 What was the total number of livestock lost by the household in the last 12 months?

Total number		N/A	

2.1.2 What were the numbers of each type of livestock lost by the household in the last 12 months?

Cattle	Sheep/goats	Horses/mules	Yaks/yak hybrids	Other	N/A

2.1.3 What were the most important reasons for these household livestock losses? RANK WITH 1 BEING THE MOST IMPORTANT.

Disease	Weather	Snow leopards	Other predators	Theft	Accident	Other	N/A

2.1.4 What was the total number of household livestock killed by snow leopards in the last 12 months?

Total number		N/A	

2.1.5 What were the numbers of each type of household livestock killed by snow leopards in the last 12 months?

Cattle	Sheep/goats	Horses/mules	Yaks/yak hybrids	Other	N/A

2.1.6 Where were the main locations of these livestock killings by snow leopards? RANK WITH 1 BEING THE MOST IMPORTANT.

High pastures	Low pastures	Barren land	Agriculture/ settlement	Scrubland	Other	N/A

2.1.7 Which were the main months when most of these livestock killings by snow leopards took place? RANK WITH 1 BEING THE MOST COMMON.

Jan/ Feb	Feb/ Mar	Mar/ Apr	Apr/ May	May/ Jun	Jun /July	Jul/ Aug	Aug/ Sep	Sep / Oct	Oct/ Nov	Nov/ Dec	Dec/ Jan	Not sure	N/A
Magh	Falgun	Chaitra	Baisakh	Abhishek	Asar	Shrawan	Bhadau	Asoj	Kartik	Mangsir	Poush	---	---

2.1.8 Did your household receive compensation for the livestock killed by snow leopards?

Yes		No		Not yet		N/A	

2.1.9 Positive identification of snow leopard?

Yes		No		N/A	

2.1.10 Positive differentiation between snow leopard and common leopard?

Yes		No		N/A	

2.2 Conflict with snow leopard conservation

Has your household had a conflict with any of the following organisations involved in snow leopard conservation in the last 12 months and, if so, why was this?

		Yes	No	N/A
2.2.1	Park management			
2.2.2	Park management			
2.2.3	Local committee			
2.2.4	Local committee			

Has your household had a conflict with any of the following snow leopard conservation measures in the last 12 months and, if so, why was this?

		Yes	No	N/A
2.2.5	Ban on the killing of snow leopards			
2.2.6	Ban on the killing of snow leopards			
2.2.7	Ban on the killing of snow leopard prey			
2.2.8	Ban on the killing of snow leopard prey			
2.2.9	Livestock compensation scheme			
2.2.10	Livestock compensation scheme			
2.2.11	Corral construction			
2.2.12	Corral construction			
2.2.13	Environmental education activities			
2.2.14	Environmental education activities			
2.2.15	Limits on the collection of NTFPs			
2.2.16	Limits on the collection of NTFPs			

2.2.17	Limits on the collection of wood			
2.2.18	Limits on the collection of wood			
2.2.19	Other			
2.2.20	Other			

2.2.21 If other, please state what.

Section 3 Individual attitudes

3.1 Respondent attributes
3.1.1 What is your age?

3.1.2 What is your gender?

Male		Female	

3.1.3 How many years of education have you had?

3.1.4 Are you a native of the area?

Yes		No		N/A	

3.1.5 What is your religion?

None		Buddhist		Bon		Hindu		Other		N/A	

3.1.6 How religious would you define yourself?

Very religious	Quite religious	Neutral	Not very religious	Not religious at all
1	2	3	4	5

3.2 Attitudes to snow leopards

3.2.1 How do you feel about snow leopards?

Very positive	Positive	Neutral	Negative	Very negative
1	2	3	4	5

3.2.2 Why do you feel this way about snow leopards?

3.2.3 Should snow leopards be present in your area in the future?

Completely agree	Agree	Neutral	Disagree	Completely disagree
1	2	3	4	5

3.2.4 Why do you feel this way about the future presence of snow leopards in your area?

3.3 Attitudes to snow leopard conservation

What is your attitude to the following organisations involved in snow leopard conservation and why do you feel this way?

		Very +ve	+ve	Neutral	-ve	Very -ve	N/A
3.3.1	Park management	1	2	3	4	5	
3.3.2	Park management						
3.3.3	Local committee	1	2	3	4	5	
3.3.4	Local conservation						

How do you feel about the following snow leopard conservation measures and why do you feel this way?

		Very +ve	+ve	Neutral	-ve	Very -ve	N/A
3.3.5	Ban on the killing of snow leopards	1	2	3	4	5	
3.3.6	Ban on the killing of snow leopards						
3.3.7	Ban on the killing of snow leopard prey	1	2	3	4	5	
3.3.8	Ban on the killing of snow leopard prey						
3.3.9	Livestock compensation scheme	1	2	3	4	5	
3.3.10	Livestock compensation scheme						
3.3.11	Corral construction	1	2	3	4	5	
3.3.12	Corral construction						
3.3.13	Environmental education activities	1	2	3	4	5	
3.3.14	Environmental education activities						
3.3.15	Limits on the collection of NTFPs	1	2	3	4	5	
3.3.16	Limits on the collection of NTFPs						
3.3.17	Limits on the collection of wood	1	2	3	4	5	
3.3.18	Limits on the collection of wood						
3.3.19	Other	1	2	3	4	5	
3.3.20	Other						

3.3.21 If other, please state what.

