# Supplementary material for: What factors best explain attitudes to snow leopards in the Nepal Himalayas?
Source: PLoS One. 2019 Oct 23;14(10):e0223565. doi: 10.1371/journal.pone.0223565 (PMC6808326; doi:10.1371/journal.pone.0223565)
Supplement: S1 Table — (DOCX) [file pone.0223565.s002.docx]

**Table S1.** Sustainable livelihood index variables and weighting

| **Asset section** | **Section weight** | **Variable name** | **Questionnaire data type** | **Index data type** | **Questionnaire number(s)** |
| --- | --- | --- | --- | --- | --- |
| Human | 20% | Adult literacy rate | Ratio | Ratio | 1.2.2 |
|  |  | School attendance rate | Ratio | Ratio | 1.2.3 |
|  |  | Medical treatment access | Ordinal | Categorical | 1.2.4 - 7 |
|  |  | Media access | Ordinal | Categorical | 1.2.8 - 11 |
| Natural | 20% | Grazing land access | Ordinal | Categorical | 1.3.1 |
|  |  | Livestock access | Continuous | Categorical | 1.3.2 - 6 |
|  |  | Cultivatable land access | Ordinal | Categorical | 1.3.7 |
|  |  | Natural products access | Ordinal | Categorical | 1.3.9 -13 |
|  |  | Water access | Ordinal | Categorical | 1.3.14 - 18 |
| Social | 20% | Formal organisation membership | Ordinal | Categorical | 1.4.1 - 9 |
|  |  | Political representatives access | Ordinal | Categorical | 1.4.10 - 12 |
| Physical | 20% | Fuel access | Ordinal | Categorical | 1.5.1 - 6 |
|  |  | Buildings access | Ordinal | Categorical | 1.5.7 - 10 |
|  |  | Transport access | Ordinal | Categorical | 1.5.11 - 17 |
| Financial | 20% | Household income | Categorical | Categorical | 1.6.1 |
