# Supplementary material for: What factors best explain attitudes to snow leopards in the Nepal Himalayas?
Source: PLoS One. 2019 Oct 23;14(10):e0223565. doi: 10.1371/journal.pone.0223565 (PMC6808326; doi:10.1371/journal.pone.0223565)
Supplement: S2 Table — (DOCX) [file pone.0223565.s003.docx]

**Table S2.** Snow leopard attitudinal scale variables and weighting

| **Variable weight** | **Variable name** | **Questionnaire data type** | **Scale data type** | **Questionnaire number** |
| --- | --- | --- | --- | --- |
| 50% | Attitudes to snow leopards | Categorical | Continuous | 3.2.1 |
| 50% | Preference for future presence of snow leopards | Categorical | Continuous | 3.2.3 |
