# Supplementary material for: What factors best explain attitudes to snow leopards in the Nepal Himalayas?
Source: PLoS One. 2019 Oct 23;14(10):e0223565. doi: 10.1371/journal.pone.0223565 (PMC6808326; doi:10.1371/journal.pone.0223565)
Supplement: S3 Table — (DOCX) [file pone.0223565.s004.docx]

**Table S3.** Snow leopard conservation attitudinal scale variables and weighting

| **Section** | **Variable weight** | **Variable name** | **Questionnaire data type** | **Scale data type** | **Questionnaire number** |
| --- | --- | --- | --- | --- | --- |
| Organisations involved in snow leopard conservation | 11.11% | Park management | Categorical | Continuous | 3.3.1 |
|  | 11.11% | Local conservation committee | Categorical | Continuous | 3.3.3 |
| Snow leopard conservation measures | 11.11% | Ban on the killing of snow leopards | Categorical | Continuous | 3.3.5 |
|  | 11.11% | Ban on the killing of snow leopard prey | Categorical | Continuous | 3.3.7 |
|  | 11.11% | Livestock compensation scheme | Categorical | Continuous | 3.3.9 |
|  | 11.11% | Corral construction | Categorical | Continuous | 3.3.11 |
|  | 11.11% | Environmental education activities | Categorical | Continuous | 3.3.13 |
|  | 11.11% | Limits on the collection of Non-Timber Forest Products | Categorical | Continuous | 3.3.15 |
|  | 11.11% | Limits on the collection of wood | Categorical | Continuous | 3.3.17 |
